# Liraglutide Attenuates Nonalcoholic Fatty Liver Disease through Adjusting Lipid Metabolism via SHP1/AMPK Signaling Pathway

**DOI:** 10.1155/2019/1567095

**Published:** 2019-05-19

**Authors:** Peng Yu, Xi Xu, Jing Zhang, Xuan Xia, Fen Xu, Jianping Weng, Xiaoyang Lai, Yunfeng Shen

**Affiliations:** ^1^Department of Endocrinology and Metabolism, Jiangxi Institute of Endocrine and Metabolic Diseases, The Second Affiliated Hospital of Nanchang University, Nanchang, China; ^2^Department of Endocrinology and Metabolism, Zhongshan Hospital, Fudan University, Shanghai, China; ^3^Department of Anesthesiology, The Second Affiliated Hospital of Nanchang University, Nanchang, China; ^4^Department of Physiology and Pathophysiology, College of Medical Sciences, China Three Gorges University, Yichang, Hubei, China; ^5^Department of Endocrinology and Metabolism, Third Affiliated Hospital of Sun Yat-Sen University, and Guangdong Provincial Key Laboratory of Diabetology, Guangzhou, China

## Abstract

A glucagon-like peptide-1 (GLP-1) receptor agonist liraglutide (LR) had been experimentally and clinically shown to ameliorate nonalcoholic fatty liver disease (NAFLD). This study aimed to investigate the beneficial effect of LR on NAFLD* in vivo* and* in vitro* and its underlying molecular mechanism. The effects of LR were examined on the high-fat diet-induced* in vivo* model in mice and* in vitro* model of NAFLD in human HepG2 cells. Liver tissues and HepG2 cells were procured for measuring lipid metabolism, histological examination, and western blot analysis. LR administration significantly lowered the serum lipid profile and lipid disposition* in vitro* and* in vivo* because of the altered expression of enzymes on hepatic gluconeogenesis and lipid metabolism. Moreover, LR significantly decreased Src homology region 2 domain-containing phosphatase-1 (SHP1) and then increased the expression of phosphorylated-AMP-activated protein kinase (p-AMPK). However, the overexpression of SHP1 mediated by lentivirus vector reversed LR-induced improvement in lipid deposition. Moreover, SHP1 silencing could further increase the expression of p-AMPK to ameliorate lipid metabolism and relative lipogenic gene induced by LR. In addition, abrogation of AMPK by Compound C eliminated the protective effects of LR on lipid metabolism without changing the expression of SHP1. LR markedly prevented NAFLD through adjusting lipid metabolism via SHP1/AMPK signaling pathway.

## 1. Introduction

Nonalcoholic fatty liver disease (NAFLD) affects millions of people worldwide. NAFLD includes diseases ranging from simple steatosis (triglyceride accumulation) to nonalcoholic steatohepatitis (NASH), advanced steatofibrosis, and even cirrhosis and ultimately hepatocellular carcinoma [1, 2]. It is strongly associated with insulin resistance, metabolic syndrome, obesity, and diabetes [3]. At present, besides lifestyle intervention and weight loss, no standard therapeutic strategies exist for simple hepatic steatosis [4]. Therefore, new effective pharmacologic treatment for NAFLD is urgently needed.

Liraglutide (LR), a glucagon-like peptide-1 (GLP-1) analog with 97% sequence identification with native human GLP-1 is widely known for treating diabetes by slowing gastric emptying, reducing food intake, potentiating glucose-dependent insulin secretion, and stimulating pancreatic beta-cell growth [5]. Over the past few years, LR has shown to be promising in treating patients with diabetes with or without NAFLD [6–8]. It could reduce the HbA1c and intrahepatic fat (IHF) content and recover the liver function in patients with type 2 diabetes mellitus (T2DM) with NAFLD [9]. Moreover, LR could also prevent fibrosis in patients with NASH [10]. However, the precise molecular mechanisms underlying the effect of LR on NAFLD are still poorly understood.

AMP-activated protein kinase (AMPK), a conserved serine/threonine protein kinase, is an energy sensor that regulates hepatic lipid metabolism [11]. AMPK has become a potential therapeutic target for treating NAFLD because of its important role in the control of lipid accumulation in hepatocytes. He et al. reported that LR exerted protective effects against NAFLD* in vitro*, which might be partially mediated by the AMPK/mTOR pathway [12]. Furthermore, LR was shown to reduce fatty degeneration induced by free fatty acids (FFAs) in hepatocytes through the AMPK/sterol regulatory element-binding protein 1c (SREBP-1c) pathway [13]. Therefore, LR-induced AMPK activation seems to play a vital role in improving hepatocyte steatosis.

The protein tyrosine phosphatase (PTP) family can be broadly divided into two major groups based on their substrate specificity: transmembrane receptor-like proteins and nontransmembrane or nonreceptor cytoplasmic PTPs [14]. Nontransmembrane or nonreceptor PTPs contain two tandem Src homology region 2 domains, defined as SHP1 and SHP2 [14]. The cytoplasmic PTPs, SHP1, and SHP2 are vital in the control of numerous signaling pathways to regulate immune response and fundamental physiological processes [14–16]. The SHP1 protein, also named PTP nonreceptor type 6 (PTPN6), is expressed primarily in most epithelial cells, but has also been detected in liver and skeletal muscle [17]. Previous studies demonstrated that SHP1 was generally recognized as a key modulator of insulin resistance and glucose metabolism in the liver, which participated in the pathogenesis of NAFLD as a negative regulator [18, 19]. Furthermore, the “knockdown” of SHP1 by small hairpin RNA in hepatocytes showed increased insulin sensitivity and glucose tolerance [19, 20]. Concomitantly, a recent study using liver-specific SHP1 KO mice showed that these mice were protected from obesity-induced severe NAFLD, which was possibly a result of reduced PPAR*γ* promoting insulin resistance and hepatic inflammation [18]. Regarding lipid oxidation and metabolism in the liver, an SHP1 deficiency also prevented the accumulation of hepatic lipids and the development of severe inflammation and oxidative stress in HFD-induced steatotic livers [19]. Intriguingly, no study has reported on the involvement of SHP1/AMPK pathway in the pathogenesis of NAFLD. However, to investigate the specific association between SHP1 and AMPK in NAFLD, it is essential to explore the effect of the SHP1/AMPK pathway on LR-induced improvement in NAFLD.

The present study investigated the association between LR, hepatic steatosis, and the SHP1/AMPK pathway. This study for the first time revealed a significant lipid-lowering effect of LR on both* in vivo* and* in vitro* NAFLD models. It also found that treatment with LR alleviated the hepatic lipid accumulation in the rat liver and cultured hepatocytes and that the underlying molecular mechanisms might be associated with the suppression of SHP1 and the activation of AMPK pathway.

## 2. Materials and Methods

### 2.1. Animal Experiments

All animal protocol was approved by the animal care and ethics committee of Second Affiliated Hospital of Nanchang University and carried out in accordance with the National Institutes of Health Guide (NIH publication no. 85-23 revised 1985) for the care and use of laboratory animals. All mice were housed in a 12-h light/12 h dark cycle with 50%–60% humidity at 21°C–23°C.

An SHP1 knockdown mouse model was constructed with these mice by administering a tail vein injection of lentivirus (LV) expressing short hairpin RNA targeting SHP1 (LV-shSHP1), which was designed and chemically synthesized by GeneChem Co. Ltd. (Shanghai, China). For the overexpression mouse experiment, LV-SHP1 LV was administered via vein injection. The control group was administered the empty vector (LV-CTL). All blood and tissue samples were rapidly centrifuged, freshly frozen in liquid nitrogen, and then stored at −80°C for the follow-up experiments. During this period, the body weight (BW) and blood glucose were monitored weekly. After sacrificing the animals, the liver of each mouse was weighed and the ratio of liver weight (LW)/BW was calculated.

### 2.2. Experimental Mouse Model of NAFLD

Twenty-five male C57BL/6J mice (aged, 8.12 ± 0.53 weeks) were used in this study. Five wild-type littermates were fed a normal chow diet (NCD). After 4 weeks of high-fat diet (HFD) (Diet Research; Nanjing, China), the mice were administered three different doses of LR (LR-L: 75 *μ*g/kg, LR-M: 150 *μ*g/kg, and LR-H: 300 *μ*g/kg; Novo Nordisk, Nanchang, Denmark) or subcutaneous injections of phosphate-buffered saline (PBS) as a vehicle control (Veh).

The HFD-fed mice were randomly divided into five groups: HFD (*n* = 5), HFD + Veh (*n* = 5), HFD + LR + LV-CTL (*n* = 5), HFD + LR + LV-shSHP1 (*n* = 5), and HFD + LR + LV-SHP1 (*n* = 5). Two weeks after the delivery of LV (5 × 10^7^ transfection units), the mice were killed in a fasting state using chloral hydrate anesthesia.

### 2.3. Cell Culture and Small Interfering RNA Transfection

Human HepG2 hepatocytes were cultured in Dulbecco's modified Eagle's medium supplemented with 10% fetal bovine serum (Gibco, MD, USA), 1% penicillin, and streptomycin (Gibco) at 37°C with 5% CO2 and 95% humidity. Stock solutions of 5 mM palmitic acid (PA; Sigma–Aldrich, MO, USA) and 5% BSA were dissolved in NaOH under appropriate heating conditions to obtain the PA-BSA complex. The PA-BSA complex was added to the culture medium to final concentration of 250*μ*M. LR was diluted to concentrations of 10 nM, 50 nM, and 100 nM for 12 h.

For SHP1 knockdown and overexpression* in vitro*, HepG2 cells were transfected with shCTL, shSHP1, LV-Green fluorescent protein (GFP), or LV-SHP1 according to the manufacturer's instructions for 48 h. After the transfection, the cells were subsequently incubated in a medium containing PA with or without 100 nM LR for 12 h. Compound C (Calbiochem, CA, USA) was used at a concentration of 20*μ*M for 12 h.

### 2.4. Histology

#### 2.4.1. Hematoxylin and Eosin Staining

Liver specimens were fixed in 4% paraformaldehyde and embedded in paraffin. Paraffin-embedded liver slices were sectioned (8 *μ*m thick) and then stained with hematoxylin and eosin (H&E). All slices were observed for pathological changes under an optical microscope (TE200; Nikon Corp, Tokyo, Japan).

#### 2.4.2. Oil Red O Staining

Fresh liver specimens were embedded in optimum cutting temperature compound (Tissue-Tek cryomold, CA, USA) and rapidly frozen sectioned into slices. Hepatocytes were fixed in 10% formaldehyde for 10 min and then washed with PBS. The frozen slices and the cells were stained using the Oil Red O solution (60% Oil Red O dye and 40% water) at room temperature for 1 h. Then, the tissue sections or cell coverslips were observed under a microscope and further quantified using the ImageJ software for detecting hepatic lipid accumulation.

### 2.5. Blood Biochemistry

Fasting blood glucose was determined in the tail vein blood using a portable glucometer (Roche, Basel, Switzerland). The serum levels of triglyceride (TG), alanine aminotransferase (ALT), aspartate transaminase (AST), and gamma-glutamyl transferase (GGT) were determined using an automatic biochemistry analyzer. Serum insulin was measured using a commercial kit (FFAs assay kit, Nanjing JianCheng Bioengineering Institute, China).

### 2.6. Western Blot

The cytoplasmic proteins of liver tissues and hepatocytes were extracted according to the instructions of the cytoplasmic protein extraction kit (Beyotime, Beijing, China). A total of 30–60 *μ*g cytoplasmic protein was resolved on an 8%–12% precast gel using sodium dodecyl sulfate–polyacrylamide* gel* electrophoresis (Invitrogen, NY, USA) and transferred to polyvinylidene fluoride membranes (Bio-Rad, CA, USA). The membranes were then incubated with anti-p-AMPK, anti-AMPK, anti-G6Pase, anti-PEPCK, anti-ACC, anti-p-ACC, anti-FAS, anti-CPT-1a, anti-SHP1, anti-SREBP-1c (Santa Cruz, CA, USA), and anti-Glyceraldehyde 3-phosphate dehydrogenase (GAPDH, Cell Signaling, MA, USA). Then, these membranes were washed with PBS and incubated with anti-mouse or anti-rabbit secondary antibody (Beyotime) for 1 h at room temperature. Finally, the immune complexes were developed using an enhanced chemiluminescence western blotting substrate, and protein expression levels were quantified using ImageJ software.

### 2.7. RT- (Real-Time-) PCR

Analysis of mRNAs was detected by quantitative RT-PCR. Total RNA was extracted from HepG2 cells using Trizol reagent and RT-PCR assays were performed as described [16]. Primers for SHP1 and *β*-actin were listed in Table S1 and *β*-actin served as a control.

### 2.8. Statistical Analysis

All data were expressed as mean ± standard deviation. Between-group comparisons were performed using the Student *t*-test. The significant difference was statistically analyzed using analysis of variance in more than two groups with the post hoc test (Tukey's multiple comparison test). Statistical significance was set at* P* < 0.05.

## 3. Results

### 3.1. LR Improved Hepatic Lipid Accumulation, Metabolic Disorder, and Liver Function in HFD-Induced Mice Models of NAFLD

**Figure 1:**
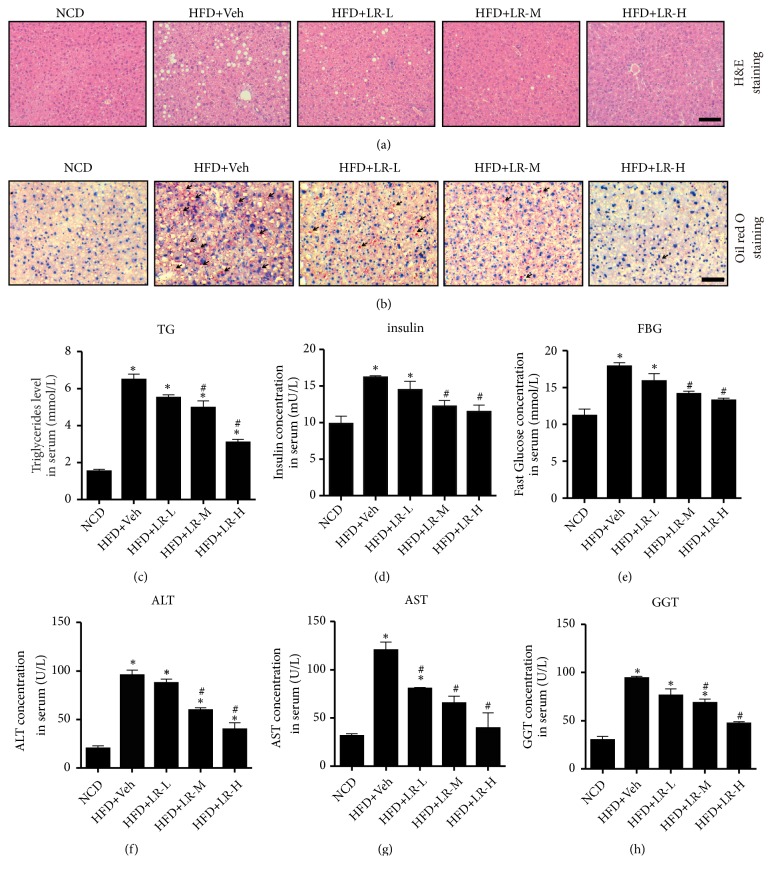
*Liraglutide improved hepatic lipid accumulation, metabolic disorder, and liver function in HFD-induced mice model of NAFLD*. (a) Liver histology was observed using hematoxylin and eosin staining. The representative images were shown (magnification ×200, scale 20 *μ*m). (b) Hepatic lipid accumulation was observed using Oil Red O staining. The representative images were shown (magnification ×200, scale 20 *μ*m). Black arrows indicated lipid droplets. (c, d, e) Metabolic parameters determined by TG, FBG, and insulin concentrations (mmol/L) in serum. (f, g, h) Liver function determined by ALT, AST, and GGT concentrations (U/L) in serum. All serous results presented as means ± SD from six independent experiments. ⁎ denotes* P* < 0.05, versus the NCD group; # denotes* P* < 0.05 versus the HFD+Veh group.

Using the HFD-induced mice model of NAFLD, the effect of LR (different concentrations: 75 *μ*g/kg, LR-L; 150 *μ*g/kg, LR-M; 300 *μ*g/kg, LR-M) in ameliorating hepatic lipid accumulation was further assessed. As expected, 4 weeks of LR injection significantly reduced BW, LV, and BW/LV (data not shown). As shown in Figure 1(a), Oil Red O staining revealed numerous lipid droplets in the livers of HFD-fed mice. However, no obvious lipid droplets were detected in the NCD group or mice treated with LR (Figure 1(b)). Remarkably, the protective effect of LR increased with the increasing dose of LR. Such changes in hepatic lipid content were confirmed by measuring serum TG levels (Figure 1(c)). The serum levels of TG significantly decreased on treatment with LR-M and LR-H (*P* < 0.05). As shown in Figures 1(d) and 1(e), administration of LR significantly improved the metabolic disorder in HFD-fed mice. The serum insulin and fasting blood glucose (FBG) levels of HFD-fed mice significantly increased compared with the NCD group, which decreased sharply after LR-M and LR-H treatment (*P* < 0.05). The liver function profile, including ALT, AST, and GGT, decreased dramatically in LR-M- and LR-H-treated HFD groups compared with the HFD + Veh group (Figures 1(f)–1(h),* P* < 0.05). No significant difference was found between LR-M and LR-H treatments. Subsequently, the intermediate concentration of LR (150 *μ*g/kg) was selected for further experiments.

### 3.2. SHP1/AMPK Pathway Was Involved in Relieving the Effects of LR on NAFLD* In Vivo*

**Figure 2:**
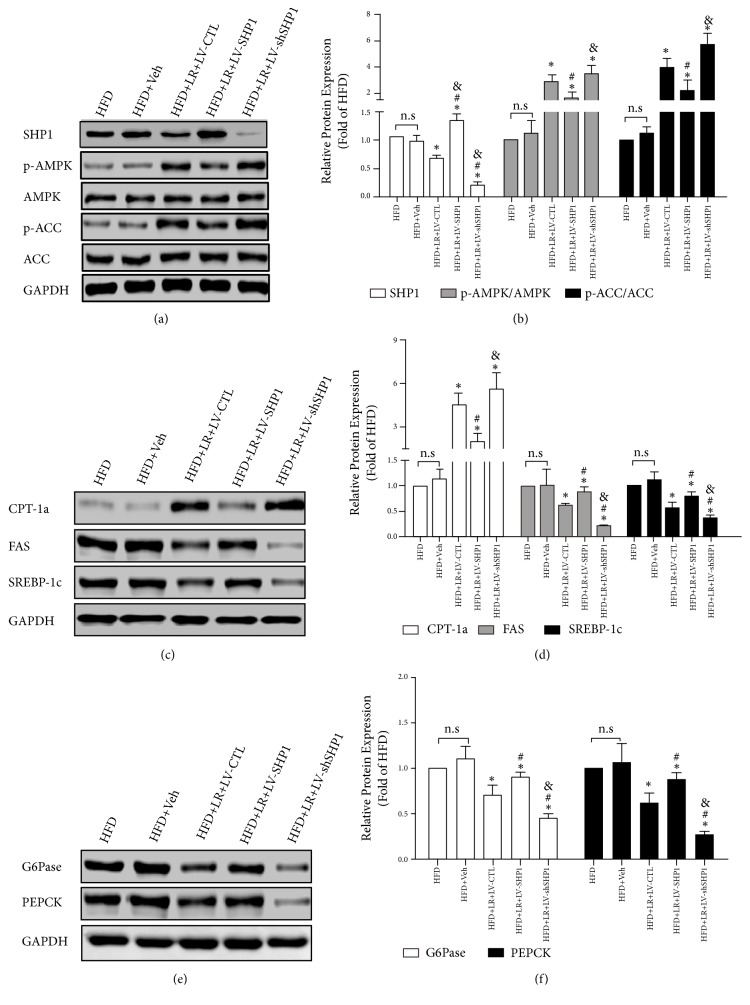
*SHP1/AMPK pathway was involved in relieving effects of liraglutide on NAFLD in vivo*. (a) The changes of levels of SHP1/AMPK pathway in all groups. SHP1, p-AMPK, AMPK, p-ACC, and ACC were detected by western blot and the representative images are shown. (b) The densitometry ratio of SHP1/GAPDH, p-AMPK/AMPK, and p-ACC/ACC are shown as mean ± SD of three independent experiments. (c) The changes of levels of lipid metabolic proteins expression in all groups. CPT-1a, FAS, and SREBP-1c were detected by western blot and the representative images are shown. (d) The densitometry ratio of CPT-1a/GAPDH, FAS/GAPDH, and SREBP-1c/GAPDH are shown as mean ± SD of three independent experiments. (e) The changes of levels of gluconeogenic proteins expression in all groups. G6Pase and PEPCK were detected by western blot and the representative images are shown. (f) The densitometry ratio of G6Pase/GAPDH and PEPCK/GAPDH is shown as mean ± SD of three independent experiments. The western blot results were normalized to the HFD group value. n.s. denotes no significance. ⁎ denotes* P* < 0.05 versus the HFD+Veh group. # denotes* P* < 0.05 versus the HFD+LR+LV-CTL group. & denotes* P* < 0.05 versus the HFD+LR+LV-SHP1 group.

Previous studies showed that the AMPK pathway was involved in relieving the effects of LR on hepatic steatosis [12, 13]. A recent study suggested that hepatocyte SHP1 might affect peroxisome proliferator–activated receptor gamma- (PPAR*γ*-) mediated hepatic inflammation by presenting obesity-induced NAFLD [15]. However, the correlation between SHP1 protein and AMPK pathway needs further exploration. Then, the expression of SHP1, AMPK, Thr172 phosphorylated AMPK [p-AMPK (T172)], SHP1, ACC, and Ser79-phosphorylated ACC [p-ACC (S79)] was detected* in vivo*. Consistent with the previous studies, the expression of SHP1 was upregulated in the liver of HFD-fed mice, while the expression of p-AMPK and p-ACC was downregulated (Figures 2(a) and 2(b)). Moreover, compared with the HFD + Veh group, LR effectively inhibited the expression of SHP1 and increased p-AMPK/AMPK and p-ACC/ACC in the HFD + LR + LV-CTL group (*P* < 0.05).

An LV injection (LV-SHP1 and LV-shSHP1) was used to genetically alter the expression of SHP1 to further investigate the cause-and-effect relationship between SHP1 and AMPK pathway, in which mice injected with LV-GFP served as controls (LV-CTL). As presented in Figures 2(a) and 2(b), compared with the HFD + LR + LV-CTL group, the overexpression of SHP1 in the liver could mildly decrease p-AMPK/AMPK and p-ACC/ACC (*P* < 0.05). On the contrary, the downregulation of SHP1 in the liver further improved the activation of AMPK pathway by LR. This was followed by increased levels of p-AMPK/AMPK and p-ACC/ACC in the HFD + LR + LV-shSHP1 group (Figures 2(a) and 2(b),* P* < 0.05). Therefore, the aforementioned results indicated that the AMPK/ACC pathway was a direct target of SHP1 protein.

### 3.3. SHP1 Played a Vital Role in LR, Increased Lipid Oxidation, and Decreased Lipogenesis and Gluconeogenesis* In Vivo*

The expression of lipid oxidative protein (ACC, p-ACC, and CPT-1), lipogenic protein (FAS and SREBP1), and gluconeogenic protein (G6Pase and PEPCK) in the liver tissue was examined to investigate the mechanism of LR action. ACC and CPT-1 are two key enzymes in the lipid entry into mitochondria and lipid oxidation in hepatocytes. As presented in Figures 2(a)–2(d), a decreased protein expression of p-ACC/ACC and CPT-1 was observed in both the HFD and HFD + Veh groups. Moreover, the ACC upstream signaling regulator AMPK also decreased as mentioned earlier. Furthermore, the present study demonstrated that LR effectively enhanced lipid oxidation in the liver. LR increased the protein expression of p-ACC/ACC and CPT-1 in the HFD + LR + LV-CTL group compared with the HFD + Veh group (Figures 2(a)–2(d),* P* < 0.05). Most importantly, the overexpression of SHP1 in the liver inhibited LR-induced lipid oxidation, and the downregulation of SHP1 further increased the lipid oxidation (Figures 2(a)–2(d),* P* < 0.05).

LR was also found to decrease the lipogenesis and gluconeogenesis in HFD-fed mice. Because FAS and SREBP1 were important proteins in lipid synthesis, the results indicated that LR reduced lipid synthesis besides increasing lipid oxidation (Figures 2(c) and 2(d),* P* < 0.05). Hepatic gluconeogenic genes were then explored in the liver tissue. LR decreased the protein expression of G6Pase and PEPCK (Figures 2(e) and 2(f),* P* < 0.05). These results suggested that LR could decrease hepatic lipid synthesis and gluconeogenesis* in vivo*.

Further studies were performed on the background of SHP1 overexpression and silencing to clarify whether the SHP1 protein mediated LR protection of lipogenesis and gluconeogenesis. On the one hand, as presented in Figures 4(c)–4(d), the protein expression levels of FAS and SREBP1 were significantly increased by LV-SHP1, and the protein expression levels of G6Pase and PEPCK were also increased by the overexpression of SHP1 (*P* < 0.05). On the other hand, as shown in Figures 4(c)–4(d), the knockdown of SHP1 further enhanced the LR-inhibited lipogenesis and gluconeogenesis. Consistently, the protein expression levels of lipogenic marker genes, including FAS and SREBP1, further decreased in the cells transfected with siSHP1 and treated with LR compared with the cells transfected with LV-CTL (Figures 2(c) and 2(d),* P* < 0.05). Subsequently, the protein expression levels of G6Pase and PEPCK further decreased in the HFD + LR + LV-shSHP1 group compared with the HFD + LR + LV-CTL group (Figures 2(e) and 2(f),* P* < 0.05). These results indicated that the SHP1/AMPK signaling pathway might be involved in the process of LR-suppressed lipogenesis and gluconeogenesis* in vivo*.

### 3.4. Overexpression of SHP1 Annihilated the Liver Protection of LR* In Vivo*

**Figure 3:**
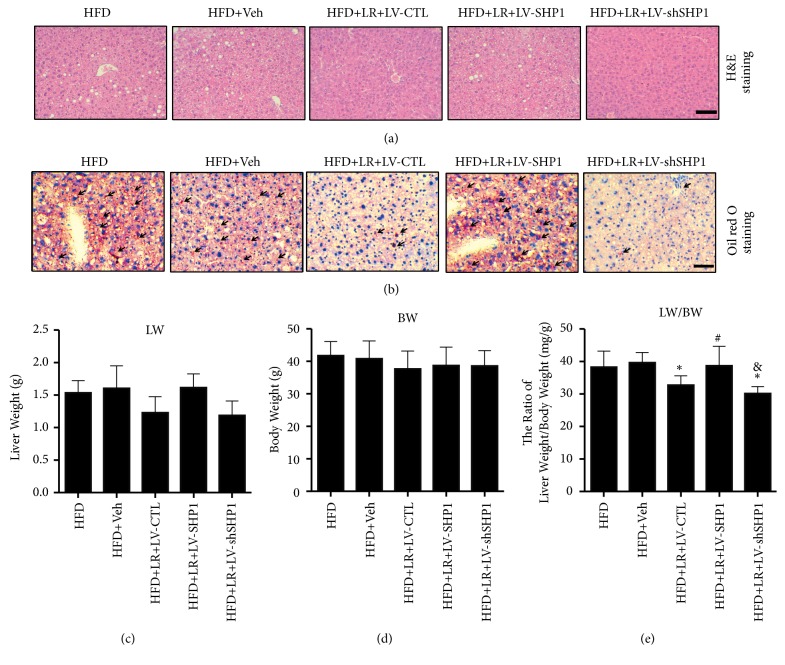
*Overexpression of SHP1 abolished the liver protection of liraglutide in vivo*. (a) Liver histology was observed using hematoxylin and eosin staining. The representative images were shown (magnification ×200, scale 20 *μ*m). (b) Hepatic lipid accumulation was observed using Oil Red O staining. The representative images were shown (magnification ×200, scale 20 *μ*m). Black arrows indicated lipid droplets. (c, d, e) The liver weight (LV) and body weight (BW) of each mouse were measured at the end of the experiments. And the ratio of LV versus BW (LV/BW) was calculated. All physiological parameters presented as means±SD from six independent experiments. ⁎ denotes* P* < 0.05 versus the HFD+Veh group. # denotes* P* < 0.05 versus the HFD+LR+LV-CTL group. & denotes* P* < 0.05 versus the HFD+LR+LV-SHP1 group.

The functional role played by SHP1 in hepatic steatosis was detected, which was the most prominent characteristic of NAFLD development. As presented in Figures 3(a) and 3(b), the H&E and Oil Red O staining analyses revealed an exacerbation of hepatic lipid accumulation caused by LV-SHP1-induced overexpression of SHP1. Consistent with the western blot analysis results, the H&E and Oil Red O staining also found that the downregulation of SHP1 markedly inhibited the hepatic lipid accumulation.

Regarding physiological parameters, no statistically significant differences were found in LW and BW between the groups (Figures 3(c) and 3(d),* P* > 0.05). However, LW and BW reduced the trend in LR-treated groups. Notably, a continuous LR for 4 weeks led to a significant decrease in the ratio of LW/BW in the HFD + LR + LV-CTL and HFD + LR + LV-shSHP1 groups compared with the HFD + Veh group (Figure 3(e),* P* < 0.05). In line with the exacerbated lipid accumulation, the overexpression of SHP1 induced a marked increase in the ratio of LW/BW in the HFD + LR + LV-SHP1 group (Figure 3(e),* P* < 0.05 versus HFD + LR + LV-CTL group).

### 3.5. SHP1/AMPK Pathway Was Involved in Relieving the Effects of LR on Hepatocellular Steatosis* In Vitro*

To clarify the* in vivo* effects of LR on the SHP1/AMPK pathway, a series of experiments were performed in cultured HepG2 cells. Using* in vitro* assay, no obvious morphological changes were found in LR-treated cells. At first, LR decreased lipid deposition in PA-treated cells, especially at concentrations of 50 and 100 nmol/L (Figure S1,* P* < 0.05). As 50 nmol/L LR had optimal effects on hepatocellular steatosis* in vitro*, this concentration of LR was used for all the following experiments. Next, whether the suppression of hepatocellular steatosis by LR in HepG2 cells was mediated via SHP1 and its downstream signaling pathway was investigated. As shown in Figure S2, LR-inhibited the mRNA and protein expression of SHP1 in PA-treated HepG2 cells (*P* < 0.05).

**Figure 4:**
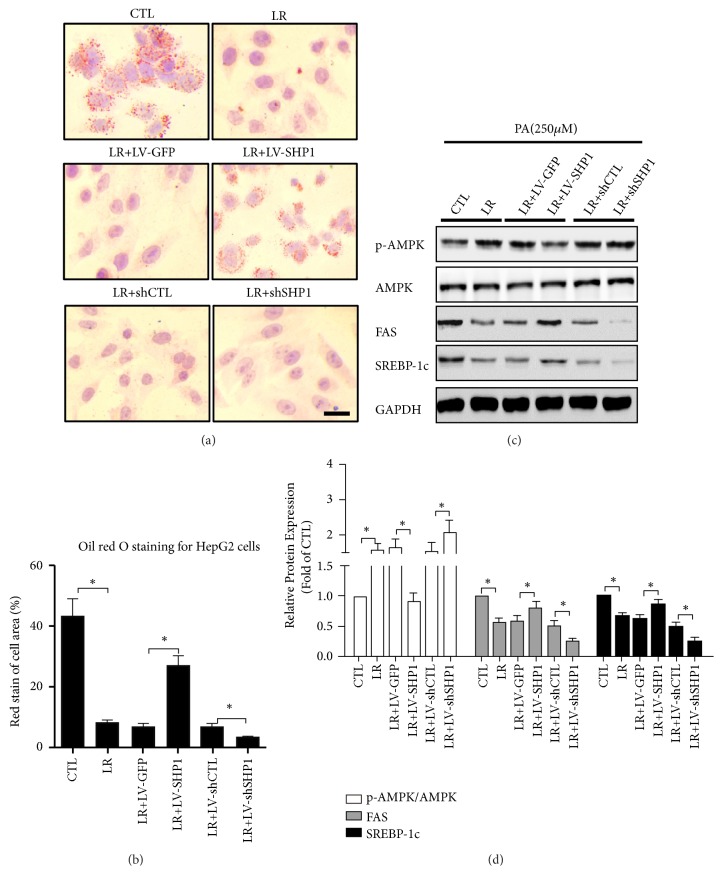
*SHP1/AMPK pathway was involved in relieving effects of liraglutide on hepatocellular steatosis in vitro*. (a) HepG2 cells were cultured in FBS-free DMEM with 250 nmol/L PA in 24 hours and then exposed to liraglutide for 24 hours after cells had been transfected by LV-SHP or control LV-GFP, shSHP, and control shCTL. And cells were stained with Oil Red O and photographed under the microscope (magnification ×200, scale 20 *μ*m). (b) Intracellular lipid accumulation was quantified by semiquantitative analysis. The rate of cell with positive area is shown as mean ± SD of three independent experiments. (c) The changes of levels of AMPK pathway proteins and lipid lipogenesis proteins in all groups. FAS, SREBP-1c, p-AMPK, AMPK, p-ACC, and ACC were detected by western blot and the representative images are shown. (d) The densitometry ratio of FAS/GAPDH, SREBP-1c/GAPDH, p-AMPK/AMPK, and p-ACC/ACC are shown as mean ± SD of three independent experiments. The western blot results were normalized to the control value. ⁎ denotes* P* < 0.05.

The SHP1 overexpression lentivirus (LV-SHP1) and SHP1 knockdown lentivirus (shSHP1) were used to change the expression of SHP1 in the cells (Figure S3,* P* < 0.05). Notably, the increase in the expression of SHP1 by LV-SHP1 annihilated the protective effects of LR on hepatocellular steatosis (Figures 4(a) and 4(b),* P* < 0.05). Moreover, the upregulation of the expression of SHP1 in hepatocytes eliminated the LR-induced activation of AMPK and the inhibition of lipogenesis, which was indicated by the decreased expression of p-AMPK/AMPK and increased expression of FAS and SREBP-1c (Figures 4(c) and 4(d),* P* < 0.05). However, after the SHP1 knockdown, the hepatocellular AMPK level was assessed in the PA-treated HepG2 cells. It was unexpectedly found that shSHP1 further increased the expression levels of p-AMPK/AMPK (Figures 4(c) and 4(d),* P* < 0.05). In parallel, the treatment of LR further decreased the expression of FAS and SREBP-1c in HepG2 cells exposed to PA (Figures 4(c) and 4(d),* P* < 0.05). These results suggested that LR could attenuate hepatocellular steatosis in hepatocytes and was dependent on the SHP1/AMPK pathway.

### 3.6. AMPK Inhibitor Blocked the LR-Induced Reduction of Lipid Lipogenesis without Changing the Expression of SHP1

**Figure 5:**
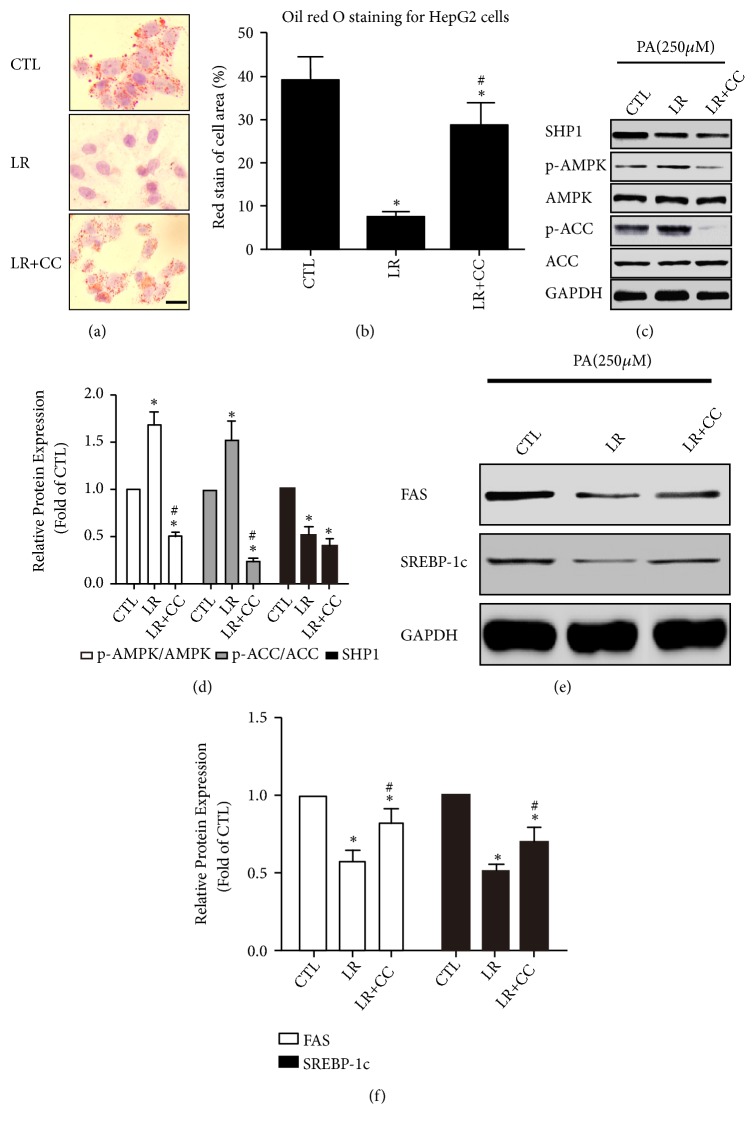
*AMPK inhibitor could block liraglutide induced reduction of lipid lipogenesis and without change on SHP1 expression*. (a) HepG2 cells were cultured in FBS-free DMEM and then subjected to 250 nmol/L PA in 24 hours and exposed to indicated drugs for 4 hours. And cells were stained with Oil Red O and photographed under the microscope (magnification ×200, scale 20 *μ*m). (b) Intracellular lipid accumulation was quantified by semiquantitative analysis. The rate of cell with positive area is shown as mean ± SD of three independent experiments. (c, d) Effect of AMPK inhibitor compound C (CC) on protein expression of SHP1, p-AMPK, AMPK, p-ACC, and ACC* in vitro*. The densitometry ratio of SHP1/GAPDH, p-AMPK/AMPK, and p-ACC/ACC are shown as mean ± SD of three independent experiments. (e, f) Effect of CC on protein expression of lipogenesis proteins (FAS and SREBP-1c)* in vitro*. The densitometry ratio of FAS/GAPDH and SREBP-1c/GAPDH is shown as mean ± SD of three independent experiments. Whole-cell protein was harvest and detected by western blot. The representative images are shown. The western blot results were normalized to the CTL group value. ⁎ denotes* P* < 0.05 versus the CTL group. # denotes* P* < 0.05 versus the LR group.

To confirm that the AMPK pathway and their downstream targets are indeed downstream of SHP1 activation, HepG2 cells were preincubated with CC before being exposed to LR. As shown in Figures 5(a) and 5(b), hepatic accumulation of lipid droplets was significantly lower in the LR group than in the CTL group (only PA-treated HepG2 cells) and increased after CC treatment (*P* < 0.05). Next, the expression levels of SHP1, p-AMPK, AMPK, p-ACC, and ACC were measured. No doubt, AMPK signaling activation was observed in the LR group, and the expression of p-AMPK and p-ACC significantly decreased in the LR + CC group than in the LR group (Figures 5(c) and 5(d),* P* < 0.05). However, LR significantly reduced the protein expression of SHP1, but not in the cells pretreated with CC (Figures 5(c) and 5(d),* P* > 0.05). Moreover, PA exposure induced a significant increase in the expression of FAS and SREBP-1c, and LR could decrease the lipogenesis and the expression of FAS and SREBP-1c (Figures 5(e) and 5(f),* P* < 0.05). Compared with the LR-treated cells, administration of CC resulted in higher rates of lipogenesis, which was indicated by the increased protein expression of FAS and SREBP-1c (Figures 5(e) and 5(f),* P* < 0.05). The treatment with CC, an AMPK inhibitor, further elucidated that LR-inhibited SHP1 and then phosphorylated AMPK to reduce lipid lipogenesis* in vitro. *

## 4. Discussion

Several epidemiological studies have revealed that weight gain greatly increases the risk and burden of hepatic steatosis and NAFLD with or without T2DM [3, 21–23]. It was reported recently that antidiabetics might become an attractive therapeutic option for treating patients with T2DM and NAFLD given their common pathophysiological features [24, 25].

Among the new kind of antidiabetics, GLP-1 receptor agonist LR is an incretin mimetic that has been shown to be promising in treating NAFLD patients with or without diabetes [6–8]. In a small randomized trial, 1.8 mg LR administered subcutaneously was effective in reducing the HbA1c and IHF content, thereby improving the liver function in patients with T2DM accompanied by NAFLD [26]. Moreover, a number of animal and cell studies showed that LR therapy improved hepatic insulin resistance and lipogenesis-induced lipid accumulation and decreased steatosis and even fibrosis [6, 12, 13, 27–29]. The results of the present study were in accordance with the previous findings, which showed a decrease in lipid accumulation and lipogenesis with LR in* in vivo* and* in vitro* experiments. This decrease was mediated by the decreased expression of SHP1 and then activation of the AMPK signaling pathway.

Notably, SHP1 is generally recognized as a key modulator of insulin action and glucose metabolism in the liver. It participates in the pathogenesis of insulin resistance and NAFLD as a negative regulator [18, 20]. Moreover, a more recent study using liver-specific SHP1 knockout (KO) mice revealed that SHP1 deficiency markedly improved obesity-linked NAFLD, which is possibly a result of reduced liver inflammation and hepatocellular damage [18]. In our study, a significant upregulation of SHP1 was observed in both HFD-fed mice and PA-treated HepG2 cells. Furthermore, this study investigated whether LR ameliorated lipid accumulation and liver steatosis dependent on SHP1 inhibition. It suggested that SHP1 inhibition was required for the effect of LR, which decreased lipid deposition in the liver by inhibiting lipogenesis and increasing lipid oxidation, as indicated by the downregulation of FAS and SREBP1. On the contrary, the overexpression of SHP1 reversed the metabolic benefits of LR on lipid metabolism. Interestingly, the overexpression of LV-SHP1-mediated SHP1 eliminated the LR-induced activation of AMPK and the alleviation of hepatic lipid accumulation* in vitro*. Therefore, the hypothesis that AMPK pathway–mediated lipogenesis is directly regulated by SHP1 was tested, and it was demonstrated that PTP negatively regulated AMPK activity in the liver. AMPK is known to be the master regulator of growth regulation and metabolism reprogramming, which suppresses lipogenesis pathways in the development of NAFLD [12, 13, 30–33]. The effect of LR in reducing fatty degeneration in hepatocytes is mediated by the AMPK/SREBP1 pathway [13]. The present study revealed that LR had a stronger effect on the activation of AMPK signaling* in vivo* and* in vitro*, which was in accordance with the previous findings. Moreover, the drug inhibition assay was performed, and it was found that blocking AMPK activation in cells by pretreatment with CC resulted in the annihilation of the reduction in lipid lipogenesis by LA without changing the expression of SHP1. This study provided sufficient evidence to believe that AMPK was the downstream protein kinase of SHP1, and SHP1/AMPK signaling pathway was important in the hepatic lipid protection of LR.

This study first confirmed that hepatic SHP1 protein increased in HFD-fed NAFLD mice models and that this increase could be reversed after disease remission by LR treatment. Moreover, the* in vivo *and* in vitro* data were consistent with those of previous studies showing that LR-suppressed the hepatic steatosis and decreased the expression of lipogenic proteins through AMPK-dependent mechanism. Remarkably, SHP1 deficiency markedly increased the activation of AMPK pathway* in vivo *and* in vitro*, protecting against NAFLD-induced liver damage.

In conclusion, the main finding of our present study was that the antilipotoxic effect of LR in the liver might be mediated by the inhibition of hepatic SHP1 and then the activation of AMPK.

## Data Availability

The data used to support the findings of this study are available from the corresponding author upon request.

## Supplementary Materials

Supplementary Materials Figure S1. Liraglutide ameliorated lipid accumulation in PA-treated hepatocytes. Figure S2. Liraglutide inhibited SHP1 mRNA and protein expression in PA-treated hepatocytes. Figure S3. Overexpression and knockdown of SHP1 in PA-treated hepatocytes. Table S1. Real-time PCR primer sequences for SHP1 and *β*-actin.
