# Supplementary material for: Liraglutide Attenuates Nonalcoholic Fatty Liver Disease through Adjusting Lipid Metabolism via SHP1/AMPK Signaling Pathway
Source: Int J Endocrinol. 2019 May 19;2019:1567095. doi: 10.1155/2019/1567095 (PMC6545813; doi:10.1155/2019/1567095)
Supplement: Supplementary Materials — Figure S1. Liraglutide ameliorated lipid accumulation in PA-treated hepatocytes. Figure S2. Liraglutide inhibited SHP1 mRNA and protein expression in PA-treated hepatocytes. Figure S3. Overexpression and knockdown of SHP1 in PA-treated hepatocytes. Table S1. Real-time PCR primer sequences for SHP1 and β-actin. [file 1567095.f1.doc]

***Supplementary figures and legends***


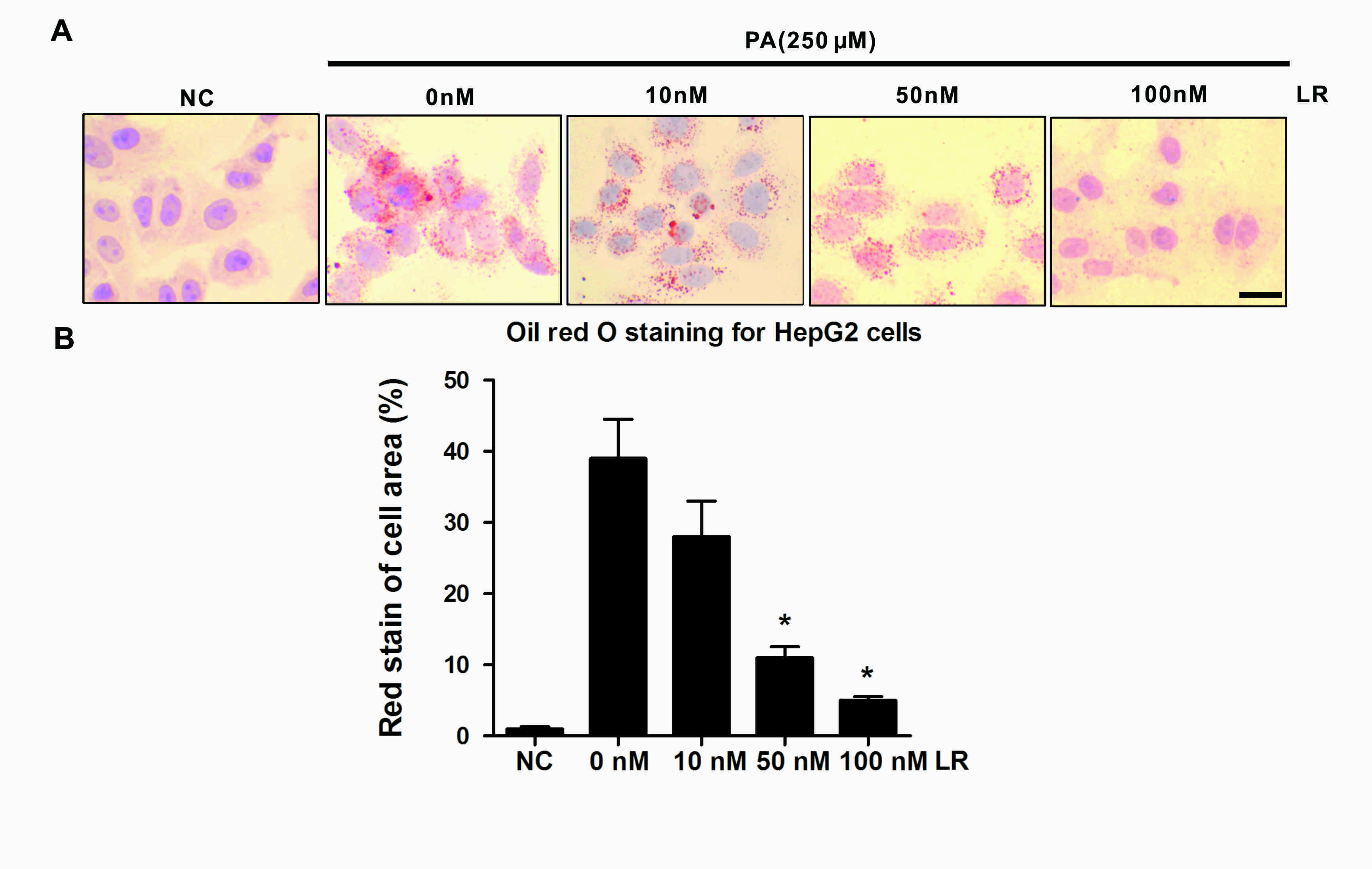


**Figure S1. Liraglutide ameliorated lipid accumulation in PA-treated hepatocytes. (A)** HepG2 cells were cultured in FBS-free DMEM, then subjected to 250umol/L PA or negative control (NC) in 24hours and indicated concentration of liraglutide (LR: 10nmol/L, 50nmol/L, 100nmol/L). And the cells were stained with Oil Red O and photographed under the microscope (magnification ×200, scale 20 μm). **(B)** Intracellular lipid accumulation was quantified by semi-quantitative analysis. The rate of cell with positive area is shown as mean ± SD of three independent experiments. All data presented as means ± SD from three independent experiments. ***** denotes *P* < 0.05.

**
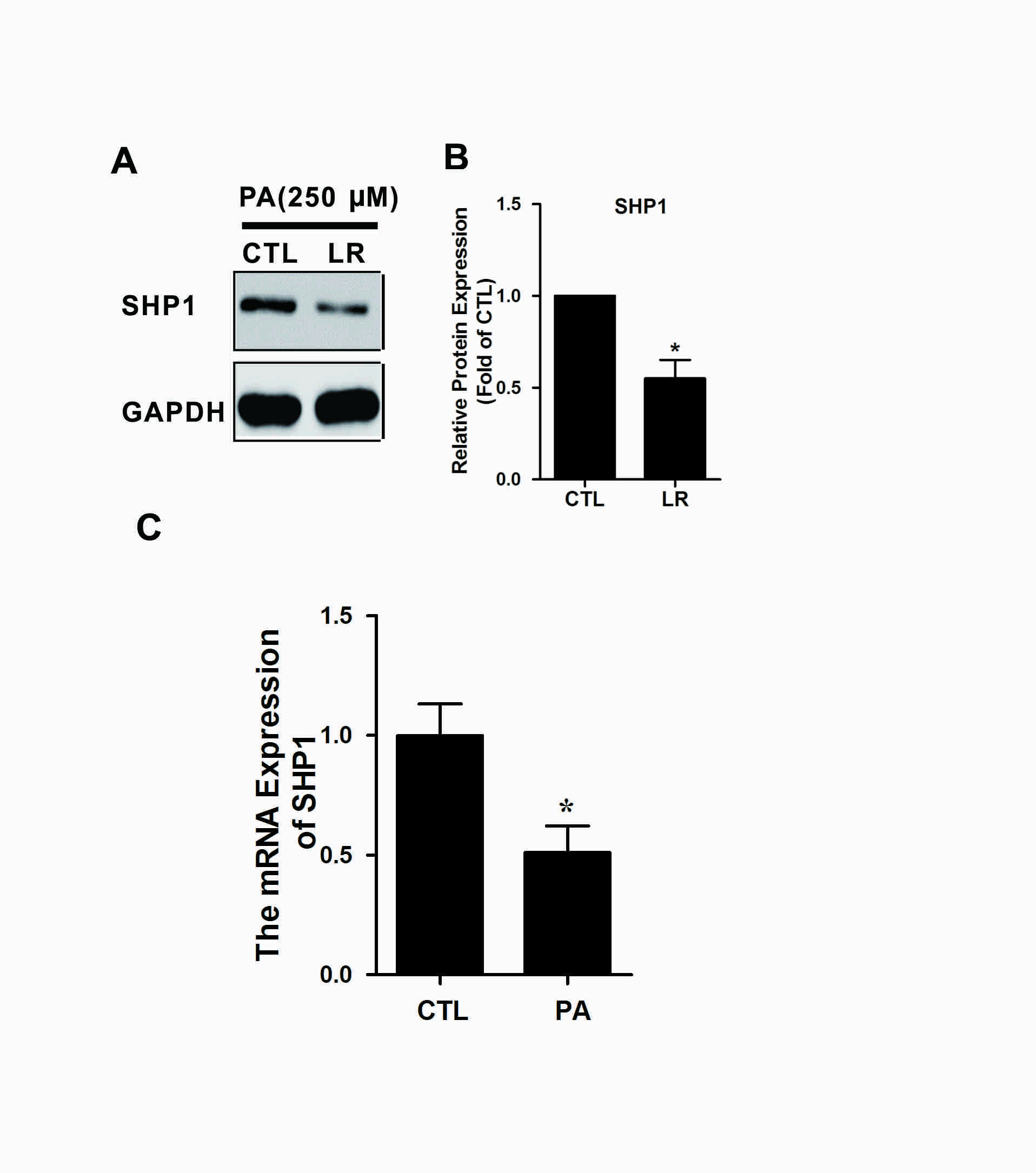
**

**Figure S2. Liraglutide inhibited SHP1 mRNA and protein expression in PA-treated hepatocytes. (A)** SHP1 protein expression with liraglutide treatment for 24hours. And the representative western blot images are shown. **(B)** The densitometry ratio of SHP1/GAPDH are shown as mean ± SD of three independent experiments. The western blot results were normalized to the control value. **(C)** Real-time PCR was performed for the analysis of the mRNA level of SHP1 (n = 6 per group). All values are the means ± SD. ***** denotes *P* < 0.05 vs Control group.

**
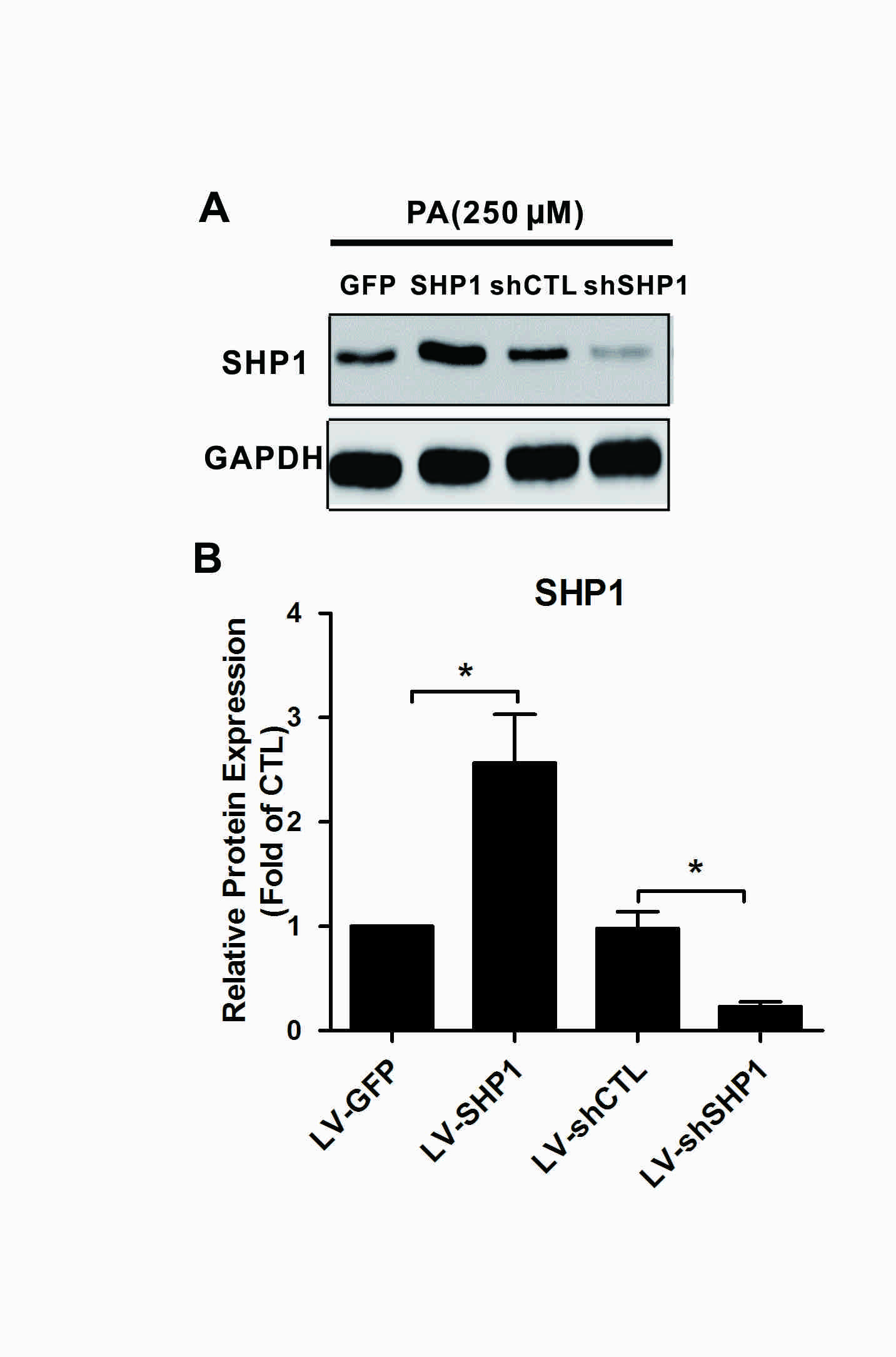
**

**Figure S3. Overexpression and knockdown of SHP1 in PA-treated hepatocytes. (A)** The protein expression of SHP1 in different groups was detected by western blot and the representative images were shown. **(B)** The densitometry ratio of SHP1/GAPDH are shown as mean ± SD of three independent experiments. The western blot results were normalized to the control value. ***** denotes *P* < 0.05. **(C)**

**Table S1. Real-time PCR** **Primer Sequences**

| **Target gene** | Sequences (5’-3’) |
| --- | --- |
| **SHP1** | forword: 5- CCGCATGTCTACTGGGCTC-3  reverse: 5- GGCAGGGTTCTGTACGTCAC-3 |
| **β-actin** | forword:5-TAAAGACCTCTATGCCAACACAGT-3  reverse:5-CACGATGGAGGGGCCGGACTCATC-3 |
